# Association between stress hyperglycemia ratio and poor outcomes in Trauma surgery ICU patients

**DOI:** 10.1371/journal.pone.0323085

**Published:** 2025-05-09

**Authors:** Heshan Cao, Junying Wei, Wuhua Ma, Yuhui Li

**Affiliations:** 1 Department of Biobank and Bioinformatics, Sun Yat-sen Memorial Hospital, Sun Yat-sen University, Guangzhou, China; 2 First Clinical Medical College, Guangzhou University of Chinese Medicine, Guangzhou, China; 3 Department of Anaesthesiology, The First Affiliated Hospital of Guangzhou University of Chinese Medicine, Guangzhou, China; University of Diyala College of Medicine, United States of America

## Abstract

**Background:**

Patients in the Trauma Surgery Intensive Care Unit (TSICU) often experience severe stress responses, which may lead to the occurrence of stress hyperglycemia. The stress hyperglycemia ratio (SHR), a biomarker quantifying the relative severity of stress hyperglycemia, has garnered increasing attention. This study aims to investigate the association between SHR and poor outcomes in TSICU patients.

**Methods:**

A retrospective cohort study was conducted based on the Medical Information Mart for Intensive Care IV database. Patients in the TSICU were stratified into tertiles based on SHR values. The primary outcomes were 30-day and 365-day all-cause mortality, and the secondary outcome was hospital mortality. Kaplan-Meier survival analysis, logistic regression, Cox proportional hazards models, and restricted cubic spline analysis were employed to examine the relationship between SHR and poor outcomes. The potential incremental value of incorporating SHR into traditional disease severity scoring systems was also explored.

**Results:**

A total of 569 eligible TSICU patients were included. The 30-day and 365-day all-cause mortality rates were 20.7% (118 patients) and 32.5% (185 patients), respectively. Higher SHR was associated with significantly increased risks of 30-day, 365-day, and hospital mortality (HR/OR > 1, *P* < 0.05). Restricted cubic spline analysis demonstrated no significant non-linear relationship between SHR and mortality risk (*P* > 0.05). Furthermore, SHR provided incremental prognostic value when integrated into traditional disease severity scoring systems.

**Conclusion:**

High SHR is significantly associated with increased all-cause mortality in TSICU patients, particularly among non-diabetic individuals. As a prognostic marker, SHR shows potential clinical utility for early risk stratification and management optimization.

## Introduction

The Trauma Surgery Intensive Care Unit (TSICU) is a specialized ward dedicated to the management of patients with traumatic injuries or those requiring acute surgical care [1,2]. TSICU patients often face severe pain and agitation, intense systemic inflammatory responses, and a high risk of complications, necessitating intensive monitoring and comprehensive therapeutic interventions [3,4]. Moreover, these patients frequently experience profound stress responses, leading to significant fluctuations in blood pressure, heart rate, and blood glucose levels, which in turn influence their treatment and prognosis [5,6].

Stress hyperglycemia (SH) refers to the acute elevation of blood glucose levels during states of severe stress, driven by complex interactions among endocrine, metabolic, and immune responses [7]. Extensive research has shown that SH is not only a crucial marker of physiological stress but is also closely associated with adverse outcomes, including increased mortality, prolonged hospital stays, and elevated infection risks [8–11]. SH may reflect disease severity through markedly elevated glucose levels or glucose variability [12]. Additionally, it may directly contribute to poor outcomes via mechanisms such as endothelial dysfunction and oxidative stress [7]. Consequently, the timely identification and effective management of SH are essential for improving patient outcomes.

Traditionally, clinicians have relied on admission blood glucose levels as a measure of SH [12]. However, since hyperglycemia at admission may stem from either acute SH or chronic diabetes-related hyperglycemia, using blood glucose levels alone makes it difficult to distinguish between these conditions. In contrast, glycated hemoglobin (HbA1c) is a well-established marker of long-term glycemic control that reflects the average blood glucose levels over the past 2–3 months, thus providing a more accurate estimate of baseline glycemic status [13]. The A1c-Derived Average Glucose Study [14] defined the estimated average blood glucose using the formula (28.7 × HbA1c [%]) - 46.7, which has been shown to correlate strongly with continuous glucose monitoring data (r² = 0.84). Building on this, Roberts et al. [15] introduced the concept of the stress hyperglycemia ratio (SHR), defined as the ratio of admission blood glucose to the estimated average glucose, thereby offering a standardized measure of the acute hyperglycemic response relative to an individual’s chronic glycemic control. Previous studies have demonstrated significant associations between SHR and adverse outcomes, including all-cause mortality, in specialized ICU settings such as neurological and cardiac ICUs [16–20]. Recently, Zhang et al. [21] explored the prognostic value of SHR in a combined cohort of surgical ICU and trauma ICU patients. However, studies focusing specifically on TSICU patients remain limited, and the role and clinical utility of SHR in this population have not been fully elucidated.

Based on the Medical Information Mart for Intensive Care IV (MIMIC-IV) database, this study aimed to investigate the relationship between SHR and the short- and long-term outcomes of TSICU patients. Furthermore, we evaluated the incremental predictive value of SHR when integrated into traditional severity scoring systems, exploring its potential clinical utility as an early risk stratification tool.

## Materials and methods

### Data source

This retrospective, observational cohort study utilized data from the MIMIC-IV (v3.1) database [22,23]. The MIMIC-IV is a publicly available, comprehensive critical care database, which includes detailed electronic medical records of over 300,000 patients treated at Beth Israel Deaconess Medical Center (BIDMC) between 2008 and 2022. The use of MIMIC data was approved by the Institutional Review Board of BIDMC (2001-P-001699/14), and all patient information in the database is fully anonymized, thus waiving the need for informed consent. The author (Heshan Cao) completed the required training and received authorization to access the database (Certification ID: 63137030).

### Study population

**Fig 1:**
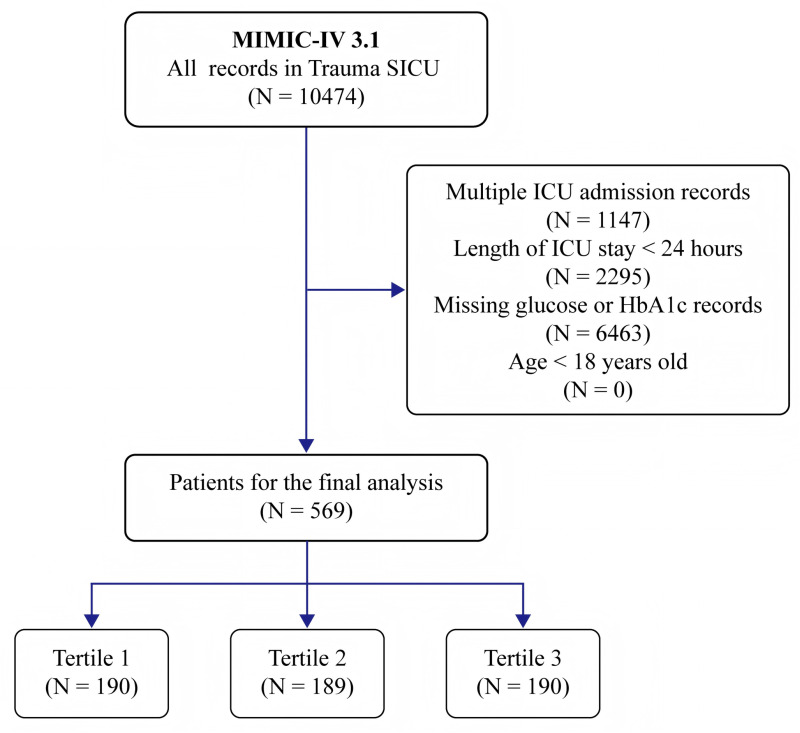
Inclusion and exclusion flowchart.

The inclusion criteria for this study comprised all adult patients admitted to the TSICU for the first time with an ICU stay exceeding 24 hours. The exclusion criteria were as follows: (1) patients younger than 18 years; (2) patients with prior ICU admissions; (3) ICU stays shorter than 24 hours; (4) missing serum glucose data within the first 24 hours of ICU admission; and (5) missing HbA1c data from the time of admission to the first three days in the ICU, ensuring that HbA1c accurately reflects average glucose levels over the preceding 8–12 weeks [24]. The inclusion and exclusion flowchart is shown in Fig 1.

### Data extraction and SHR calculation

Structured Query Language (SQL) was used to extract the following variables from the MIMIC-IV database. (1) Demographics: age, gender, race, height, weight, and body mass index (BMI); (2) Vital signs: heart rate, mean blood pressure (MBP), temperature, and pulse blood oxygen saturation (SpO2); (3) Laboratory tests: hemoglobin, platelet, anion gap, creatinine, blood urea nitrogen (BUN), sodium, potassium, and international normalized ratio (INR); (4) Comorbidities: hypertension, diabetes; (5) Medication usage: morphine, fentanyl, dexmedetomidine, antibiotic, insulin, and glucocorticoid; (6) Clinical scores: Glasgow Coma Scale (GCS), Sequential Organ Failure Assessment (SOFA), Simplified Acute Physiology Score II (SAPS II), Acute Physiology Score III (APS III), and Oxford Acute Severity of Illness Score (OASIS).

Vital signs, laboratory tests, and medication usage were collected within the first 24 hours of ICU admission. For variables with multiple measurements, the average value was used. Details on missing data are provided in S1 Fig. Variables with a missing rate exceeding 20% were excluded to minimize bias. For variables with a missing rate below 20%, multiple imputation was performed. SHR was calculated using the following formula: SHR = Admission blood glucose (mg/dL)/ (28.7 × HbA1c (%) - 46.7) [15].

### Study outcomes

The primary outcomes were 30-day and 365-day all-cause mortality. The secondary outcome was hospital mortality.

### Statistical analysis

The Kolmogorov-Smirnov test was used to assess the normality of continuous variables. Continuous variables with normal distribution were presented as means ± standard deviations (SD), while non-normally distributed variables were reported as medians (interquartile ranges, IQR). Categorical variables were presented as counts (n) and percentages (%). Differences in continuous variables were compared using Student’s t-test or Wilcoxon rank-sum test, while categorical variables were analyzed using Chi-square test or Fisher’s exact test. Multicollinearity among variables was assessed using the variance inflation factor (VIF), with a threshold of VIF > 5 indicating significant multicollinearity (S1 Table).

Based on the tertiles of SHR, patients were divided into three groups, named T1, T2, and T3, respectively, to explore the distribution characteristics of low, medium, and high levels of SHR in the study population and their relationship with clinical outcomes. Kaplan-Meier survival curves and log-rank tests were used to evaluate the association between SHR tertiles and 30-day/365-day all-cause mortality. Cox proportional hazards models were employed to estimate hazard ratios (HRs) and 95% confidence intervals (CIs) for the association between SHR and primary outcomes, analyzed as both continuous and categorical variables (with T1 as the reference). Three Cox models were developed: Model I: unadjusted; Model II: adjusted for demographics, vital signs and GCS; Model III: further adjusted for laboratory markers, comorbidities and medications. Logistic regression was used for univariate and multivariate analyses to assess the relationship between SHR and in-hospital mortality, reporting odds ratios (ORs) and 95% CIs. Restricted cubic spline (RCS) models with four knots explored the linearity or non-linearity between SHR and outcomes. Subgroup analyses by age, gender, race, weight, hypertension, and diabetes further evaluated robustness and potential interactions.

To assess the incremental prognostic value of SHR, the area under the receiver operating characteristic curve (AUC) of severity scores with and without SHR was compared using DeLong’s test. Statistical analyses were conducted using R (v4.4.1) and Python (v3.9.18). A two-tailed *P*-value < 0.05 was considered statistically significant.

## Results

### Patient characteristics

A total of 569 eligible TSICU patients were included in this study, with an average age of 63.7 years and 57.6% (328 patients) being male. Patients were stratified into three groups based on SHR tertiles: T1 (n = 190, SHR ≤ 0.968), T2 (n = 189, 0.968 < SHR ≤ 1.270), and T3 (n = 190, SHR > 1.270). Baseline characteristics indicated that patients in the high-SHR group (T3) exhibited higher heart rate, potassium, INR levels, and severity scores while being younger and having a lower proportion of White individuals (Table 1). Comparisons between T3 and T1-T2 groups revealed similar trends (S2 Table). Furthermore, S3 Table details the baseline characteristics of 30-day survivors versus non-survivors. Non-survivors had a lower proportion of White individuals and significantly higher heart rate, SpO2, anion gap, creatinine, BUN, sodium, INR, and severity scores but lower body weight and platelet counts. Notably, the SHR of non-survivors was significantly higher than that of survivors (1.28 vs. 1.08, *P* < 0.05).

**Table 1:** Baseline characteristics according to SHR tertiles.

Variables	Total (n = 569)	T1 (n = 190)	T2 (n = 189)	T3 (n = 190)	*P*-value
**Demographics**					
Age, years	66 (54, 76)	67 (55, 77)	68 (57, 77)	62 (51, 72)	<.001
Male, n(%)	328 (57.6)	105 (55.3)	108 (57.1)	115 (60.5)	0.575
Race, n(%)					0.002
White	327 (57.5)	126 (66.3)	109 (57.7)	92 (48.4)	
Other	242 (42.5)	64 (33.7)	80 (42.3)	98 (51.6)	
Weight, kg	80.5 (66.0, 97.5)	80.0 (65.6, 95.6)	81.8 (65.2, 95.6)	81.0 (68.1, 99.9)	0.264
**Vital signs**					
Heart rate, bpm	85 (74, 96)	79 (71, 90)	85 (74, 95)	89 (77, 103)	<.001
MBP, mmHg	82 (74, 90)	82 (74, 91)	83 (75, 91)	81 (74, 90)	0.433
Temperature, °C	36.9 (36.7, 37.3)	37.0 (36.7, 37.2)	37.0 (36.7, 37.3)	36.9 (36.7, 37.2)	0.300
SpO2, %	98 (96, 99)	97 (96, 99)	98 (96, 99)	98 (96, 99)	0.021
**Laboratory tests**					
Hemoglobin, g/dL	11.1 (9.4, 12.7)	11.4 (9.5, 12.9)	11.2 (9.6, 12.9)	10.8 (9.2, 12.3)	0.053
Platelet, K/μL	208 (156, 280)	220 (165, 289)	212 (154, 277)	198 (150, 264)	0.111
Anion gap, mmol/L	14 (12, 16)	14 (12, 15)	14 (12, 16)	14 (12, 16)	0.002
Creatinine, mg/dL	1.0 (0.7, 1.3)	0.9 (0.7, 1.3)	0.9 (0.8, 1.2)	1.1 (0.8, 1.5)	0.066
BUN, mg/dL	18 (13, 26)	18 (12, 24)	17 (13, 26)	19 (13, 29)	0.236
Sodium, mmol/L	139 (136, 141)	139 (137, 141)	138 (136, 141)	139 (136, 142)	0.756
Potassium, mmol/L	4.1 (3.8, 4.5)	4.1 (3.8, 4.5)	4.0 (3.7, 4.4)	4.2 (3.8, 4.8)	<.001
INR	1.2 (1.1, 1.4)	1.2 (1.1, 1.4)	1.2 (1.1, 1.3)	1.3 (1.1, 1.5)	0.019
Glucose, mg/dL	140 (113, 191)	105 (94, 128)	133 (120, 159)	200 (164, 266)	<.001
HbA1c, %	5.9 (5.5, 7.0)	6.1 (5.7, 7.8)	5.9 (5.5, 6.7)	5.7 (5.3, 6.7)	<.001
SHR	1.11 (0.89, 1.37)	0.82 (0.71,0.89)	1.11 (1.03,1.18)	1.55 (1.37,1.99)	<.001
**Comorbidities, n (%)**					
Hypertension	258 (45.3)	91 (47.9)	93 (49.2)	74 (39.0)	0.092
Diabetes	234 (41.1)	88 (46.3)	69 (36.5)	77 (40.5)	0.149
**Medications, n (%)**					
Morphine	166 (29.2)	44 (23.2)	52 (27.5)	70 (36.8)	0.011
Fentanyl	201 (35.3)	46 (24.2)	68 (36.0)	87 (45.8)	<.001
Dexmedetomidine	28 (4.9)	8 (4.2)	13 (6.9)	7 (3.7)	0.305
Antibiotic	262 (46.1)	76 (40.0)	76 (40.2)	110 (57.9)	<.001
Insulin	241 (42.4)	69 (36.3)	64 (33.9)	108 (56.8)	<.001
Glucocorticoid	77 (13.5)	25 (13.2)	23 (12.2)	29 (15.3)	0.667
**Clinical scores**					
GCS	15 (13, 15)	14 (13, 15)	14 (11, 15)	15 (14, 155)	<.001
SOFA	3 (2, 6)	3 (2, 5)	3 (2, 5)	4 (2, 7)	<.001
SAPS II	33 (26, 42)	31 (24, 41)	33 (27, 42)	36 (29, 44)	0.006
APS III	41 (31, 56)	36 (27, 49)	41 (31, 54)	46 (35, 61)	<.001
OASIS	32 (26, 37)	31 (25, 36)	32 (27, 37)	34 (27, 40)	0.006
**Outcomes, n (%)**					
30-day mortality	118 (20.7)	25 (13.2)	33 (17.5)	60 (31.6)	<0.001
365-day mortality	185 (32.5)	49 (25.8)	55 (29.1)	81 (42.6)	<0.001
Hospital mortality	97 (17.1)	23 (12.1)	25 (13.2)	49 (25.8)	<0.001

Patients were divided into T1, T2 and T3 groups according to the tertiles of SHR.

APS III, acute physiology score III; BUN, blood urea nitrogen; GCS, glasgow coma scale; HbA1c, hemoglobin A1c; INR, international normalized ratio; MBP, mean blood pressure; OASIS, oxford acute severity of illness score; SAPS II, Simplified Acute Physiology Score II; SHR, stress hyperglycemia ratio; SOFA, sequential organ failure assessment; SpO2, pulse blood oxygen saturation.

### Clinical outcomes

**Fig 2:**
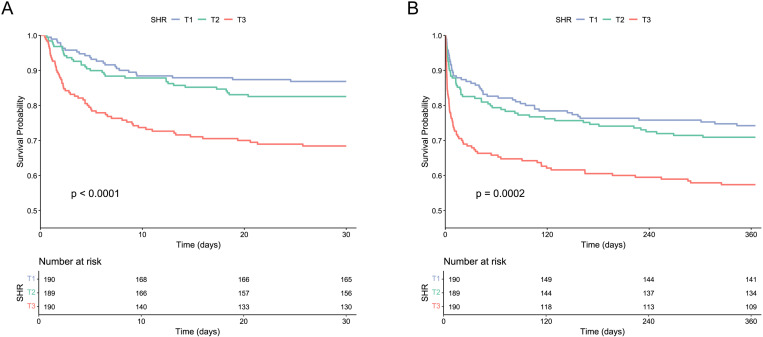
Kaplan-Meier survival analysis curves for all-cause mortality. (A) 30-day mortality. (B) 365-day mortality. Patients were divided into T1, T2 and T3 groups according to the tertiles of SHR.

**Fig 3:**
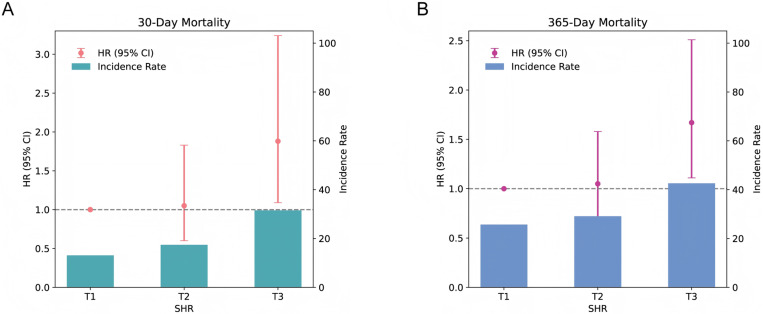
Associations of SHR with all-cause mortality. (A) 30-day mortality. (B) 365-day mortality. T1 as the reference. HR, hazard ratio; CI, confidence interval; SHR, stress hyperglycemia ratio.

**Fig 4:**
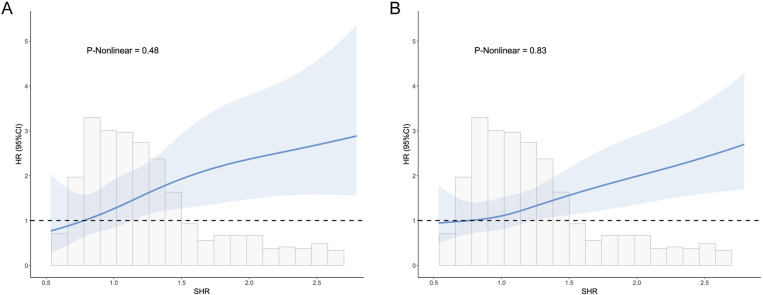
Restricted cubic spline curves of SHR with all-cause mortality. (A) 30-day mortality. (B) 365-day mortality. HR, hazard ratio; CI, confidence interval; SHR, stress hyperglycemia ratio.

The 30-day and 365-day all-cause mortality rates for the entire cohort were 20.7% (118 patients) and 32.5% (185 patients), respectively. Differential analysis revealed that the T3 group had significantly higher 30-day, 365-day, and hospital mortality compared to other groups (*P* < 0.05). Kaplan-Meier survival analysis demonstrated significant differences in 30-day and 365-day mortality across SHR tertiles (log-rank *P* < 0.05) (Fig 2). Multivariate Cox proportional hazards regression analysis, adjusted for demographics, vital signs, GCS, laboratory tests, comorbidities, and medications, confirmed that SHR, as a continuous variable, significantly associated with 30-day and 365-day all-cause mortality (Table 2). Group-based analysis indicated that the T3 group had a markedly elevated risk of mortality compared to the T1 group (Fig 3). Logistic regression analysis further confirmed a significant association between high SHR and increased hospital mortality risk (S4 Table). RCS analysis showed no significant non-linear relationship between SHR and 30-day, 365-day, and hospital mortality risks (*P* for non-linearity > 0.05) (Figs 4 and S2).

**Table 2:** Association of SHR and the risk of all-cause mortality.

Categories	Model 1		Model 2		Model 3	
**HR (95% CI)**	***P*-value**	**HR (95% CI)**	***P*-value**	**HR (95% CI)**	***P*-value**
30-day mortality						
SHR (continuous)	1.90 (1.53-2.36)	<.001	1.61 (1.25-2.08)	<.001	1.46 (1.07-1.99)	0.017
SHR (tertiles)						
T1	Ref		Ref		Ref	
T2	1.36 (0.81-2.28)	0.250	1.18 (0.70-2.00)	0.526	1.05 (0.60-1.83)	0.859
T3	2.74 (1.72-4.37)	<.001	2.08 (1.27-3.41)	0.004	1.88 (1.09-3.24)	0.023
365-day mortality						
SHR (continuous)	1.76 (1.46-2.13)	<.001	1.59 (1.28-1.98)	<.001	1.49 (1.18-1.89)	<.001
SHR (tertiles)						
T1	Ref		Ref		Ref	
T2	1.16 (0.79-1.71)	0.449	1.01 (0.69-1.49)	0.954	1.05 (0.69-1.58)	0.822
T3	1.96 (1.37-2.79)	<.001	1.62 (1.11-2.37)	0.012	1.67 (1.11-2.51)	0.015

Model 1: no adjusted.

Model 2: adjusted for demographics, vital signs and GCS.

Model 3: adjusted for demographics, vital signs, GCS, laboratory tests, comorbidities and medications.

CI, confidence interval; HR, hazard ratio; Ref, reference; SHR, stress hyperglycemia ratio.

### Subgroup analysis

**Fig 5:**
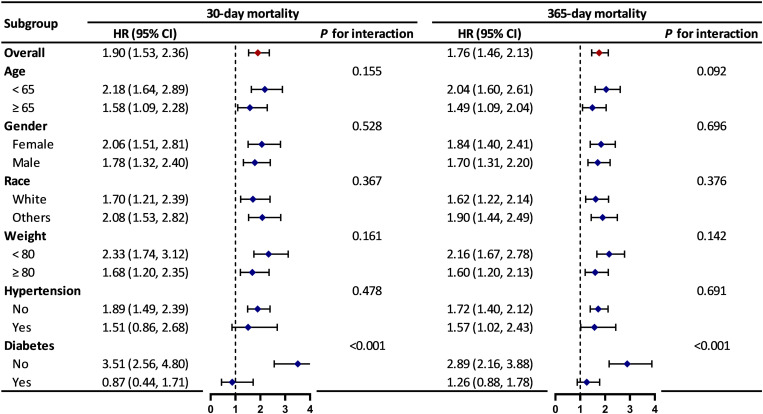
Subgroup forest plot for 30-day and 365-day mortality. HR, hazard ratio; CI, confidence interval.

Subgroup analysis stratified by age, gender, race, weight, hypertension, and diabetes demonstrated that high SHR was significantly associated with increased mortality risk in most subgroups (Fig 5). Notably, there was no significant association between SHR and increased mortality risk among patients with diabetes. Interaction analysis revealed a significant interaction between SHR and diabetes status (*P* for interaction < 0.05), with stronger associations and higher mortality risks observed for patients without a history of diabetes. These findings were robust across 30-day and 365-day mortality analyses.

### Incremental effect of SHR

**Fig 6:**
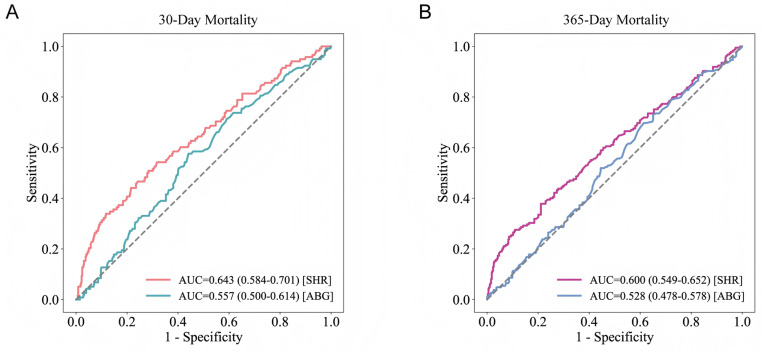
Prediction performance for 30-day and 365-day all-cause mortality by ROC curves. ABG, admission blood glucose; AUC, area under the curve; SHR, stress hyperglycemia ratio.

Using admission blood glucose alone, the AUC values for predicting 30-day and 365-day mortality were 0.557 and 0.528, respectively, whereas when using SHR as a single indicator, the AUC values increased to 0.643 and 0.600, respectively (Fig 6). The AUC values and 95% CIs of the SOFA, SAPS II, APS III, and OASIS scoring systems were calculated before and after incorporating SHR for predicting primary and secondary outcomes (Table 3, S5 Table). The results consistently showed that the inclusion of SHR improved the predictive performance of all scoring systems, with the enhancement being particularly notable for short-term mortality and hospital mortality (all DeLong test *P*-values < 0.05). For long-term mortality prediction, only the SAPS II and OASIS scores showed statistically significant performance improvements after incorporating SHR. Among all scoring systems, the combination of SOFA and SHR achieved the best performance for predicting hospital mortality in TSICU patients (AUC = 0.773).

**Table 3:** Incremental effect of SHR in predicting 30-day and 365-day mortality.

Model 1	AUC (95% CI)	Model 2	AUC (95% CI)	*P* for comparison
30-d mortality				
SOFA	0.739 (0.688-0.791)	+ SHR	0.764 (0.715-0.813)	0.007
SAPS II	0.729 (0.681-0.777)	+ SHR	0.769 (0.725-0.812)	0.002
APS III	0.732 (0.680-0.783)	+ SHR	0.759 (0.711-0.808)	0.008
OASIS	0.736 (0.688-0.784)	+ SHR	0.769 (0.723-0.816)	0.005
365-d mortality				
SOFA	0.712 (0.666-0.757)	+ SHR	0.728 (0.682-0.773)	0.050
SAPS II	0.739 (0.697-0.781)	+ SHR	0.760 (0.719-0.800)	0.020
APS III	0.717 (0.673-0.762)	+ SHR	0.732 (0.688-0.776)	0.076
OASIS	0.697 (0.651-0.743)	+ SHR	0.720 (0.675-0.766)	0.029

APS III, acute physiology score III; AUC, area under the curve; OASIS, oxford acute severity of illness score; SAPS II, simplified acute physiology score II; SHR, stress hyperglycemia ratio; SOFA, sequential organ failure assessment.

## Discussion

This study conducted a retrospective cohort analysis of 569 TSICU patients from the MIMIC-IV database to explore the relationship between the SHR and poor outcomes. Our key findings indicate that high SHR is significantly associated with increased short-term (30-day), long-term (365-day), and hospital all-cause mortality, even after adjusting for multiple confounding factors. Moreover, non-diabetic patients exhibited a notably higher risk compared to diabetic patients, and incorporating SHR into established severity scoring systems improved their predictive performance. These results underscore the important clinical value of SHR in the prognostic evaluation of TSICU patients, aiding in the early identification of high-risk patients and the optimization of management strategies.

SH refers to the significant elevation of blood glucose levels during acute stress, resulting from complex interactions among endocrine, metabolic, and immune responses [7]. In TSICU patients, trauma or surgery induces a severe stress response that activates the hypothalamic-pituitary-adrenal axis, leading to the massive release of glucocorticoids and catecholamines. These hormones promote glycogenolysis and gluconeogenesis while reducing tissue sensitivity to insulin, thereby increasing blood glucose levels [25]. In addition, inflammatory responses play a crucial role in SH. Cytokines such as TNF-α and IL-6, released during trauma, exacerbate insulin resistance, further aggravating hyperglycemia [26–28]. Elevated glucose levels not only reflect the severity of the stress response but also directly impact patient outcomes through multiple pathophysiological mechanisms. For example, hyperglycemia can impair immune function, increasing the risk of infections, and induce oxidative stress and endothelial dysfunction [29,30]. In TSICU patients, these processes exacerbate post-trauma or post-surgical multi-organ dysfunction, elevating all-cause and hospital mortality rates. Thus, SH not only serves as a marker of severe stress but may also be a critical factor driving poor outcomes.

As a marker of the relative degree of SH, SHR has gained increasing attention in recent years. This index effectively distinguishes acute stress-induced hyperglycemia from chronic diabetes-related hyperglycemia, reducing the confounding effects of baseline diabetes on research outcomes and enhancing the specificity and sensitivity of hyperglycemia as a prognostic indicator. Existing studies have demonstrated significant associations between SHR and poor outcomes across various specialized populations. For example, Xu et al. [31] identified SHR as an independent risk predictor for hospital mortality in patients with coronary artery disease. Similarly, Zeng et al. [32] explored the relationship between SHR and long-term outcomes in patients with acute coronary syndrome, finding that higher SHR levels significantly increased the risk of major adverse cardiovascular events and all-cause mortality.

In this study, we found that higher SHR levels were significantly associated with an increased risk of all-cause mortality in TSICU patients, with RCS analysis revealing a positive linear relationship between SHR and all-cause mortality risk. This finding contrasts with the U-shaped associations reported in the studies by Zhang et al. [21]. We speculate that this difference may be due to the fact that Zhang et al. focused on a combined cohort of surgical and trauma ICU patients—where TSICU patients comprised only a relatively small proportion—resulting in differences in patient selection. In our study, the linearity may reflect the unique pathophysiological state of TSICU patients, where the severe acute stress responses triggered by trauma lead to higher SHR levels that directly mirror the intensity of the response. Persistent hyperglycemia may then cause greater metabolic disruptions and immune dysfunction, continuously increasing mortality risk [8]. Nonetheless, further validation in larger-scale studies is warranted.

In the subgroup analysis, high SHR was found to be significantly associated with increased mortality risk in most subgroups, with no significant interaction effects. However, a significant interaction was observed in the diabetes subgroup (*P* for interaction < 0.05), indicating that the association between high SHR and mortality risk was stronger in patients without a history of diabetes. This finding suggests that SHR may have greater predictive value as a marker of SH in non-diabetic patients. This observation is consistent with the findings of another large-scale study [33]. A plausible explanation is that diabetic patients typically have higher baseline blood glucose levels and may have partially developed adaptive mechanisms to cope with hyperglycemia, rendering the impact of SHR variations on their prognosis relatively smaller. In contrast, in non-diabetic patients, elevated SHR during acute stress often reflects a more severe stress response and greater metabolic derangement. The absence of long-term adaptation to hyperglycemia makes their systems more susceptible to the adverse effects of high glucose levels, resulting in a higher mortality risk [34]. Furthermore, diabetic patients may receive glucose management interventions, such as insulin therapy, which could mitigate the adverse effects of hyperglycemia on their prognosis to some extent. These findings highlight the need for increased clinical attention to glucose management in non-diabetic TSICU patients to reduce their mortality risk.

The integration of SHR into traditional severity scoring systems such as SOFA, SAPS II, APS III, and OASIS consistently enhanced their predictive performance for primary and secondary outcomes, particularly for short-term and hospital all-cause mortality. This improvement is likely due to SHR functioning as an independent metabolic stress marker, capturing acute stress-induced metabolic and endocrine changes that traditional scoring systems may not adequately assess. For instance, while SOFA primarily focuses on the degree of organ failure [35], OASIS and SAPS II provide additional insights into patient severity by incorporating physiological variables and patient demographics [36,37]. In this context, SHR further elucidates the metabolic response and physiological burden during acute stress. Therefore, incorporating SHR allows for a more comprehensive evaluation of patient conditions, improving the prognostic predictive capacity of these scoring systems. The greater predictive enhancement observed for short-term and hospital mortality may stem from the more direct impact of acute stress responses on these outcomes. Persistent hyperglycemia during acute stress can rapidly impair the function of multiple organ systems, increasing the risk of severe complications and mortality in the short term [7,8]. As a relevant marker of stress response intensity, SHR can reflect the acute changes of patients and provide a more prospective risk assessment. However, the incremental effect of SHR was weaker for predicting long-term all-cause mortality, likely because long-term outcomes are influenced by a range of chronic factors, limiting the utility of a single acute stress marker like SHR in this context.

This study has several limitations. First, as a single-center, retrospective study, it may be subject to selection and information biases. Although comprehensive data from the MIMIC-IV database were used, external validation in multicenter prospective studies is needed to confirm the generalizability of our findings. Second, despite adjusting for multiple confounders, unmeasured variables such as nutritional status, specific trauma types, treatment measures, and the unknown nature of the blood glucose measurement (i.e., whether it was fasting or postprandial) may have influenced the results. These limitations may affect the interpretation of the relationship between SHR and TSICU patient outcomes. Future large-scale multicenter prospective studies are needed to validate and refine the conclusions of this research.

## Conclusion

This study found that high SHR is significantly associated with increased risks of short-term, long-term, and hospital all-cause mortality among TSICU patients, particularly in those without diabetes. As a novel biomarker for assessing poor outcomes in TSICU patients, SHR holds promising potential for clinical applications.

## Supporting information

S1 Fig Percentage of missing data in the variables included in this study. (DOCX)

S2 Fig Restricted cubic spline curve of SHR with hospital mortality. (DOCX)

S1 Table Covariance analysis between variables. (DOCX)

S2 Table Baseline characteristics according to SHR tertiles. (DOCX)

S3 Table Baseline characteristics of the 30-day survivor and 30-day mortality group. (DOCX)

S4 Table Univariate and multivariate logistic regression analysis of factors influencing hospital mortality. (DOCX)

S5 Table Incremental effect of SHR in predicting hospital mortality. (DOCX)
