# Supplementary figures and images for: Association between stress hyperglycemia ratio and poor outcomes in Trauma surgery ICU patients
Source: PLoS One. 2025 May 9;20(5):e0323085. doi: 10.1371/journal.pone.0323085 (PMC12063898; doi:10.1371/journal.pone.0323085)

**S1 Fig. Percentage of missing data in the variables included in this study.**

**
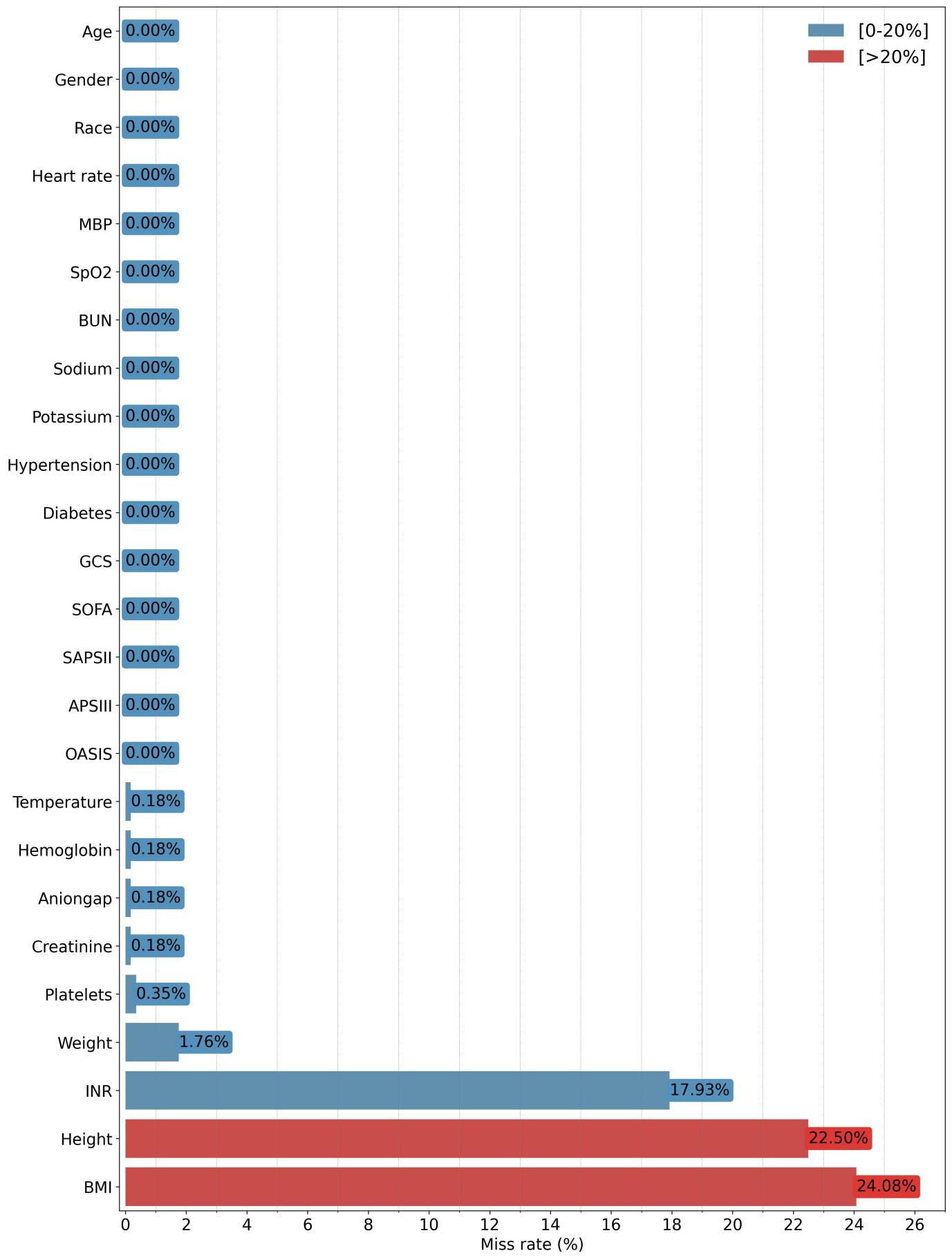
**

Supplement: S1 Fig — (DOCX) [file pone.0323085.s001.docx]

**S2 Fig . Restricted cubic spline plot of hospital mortality.**

**
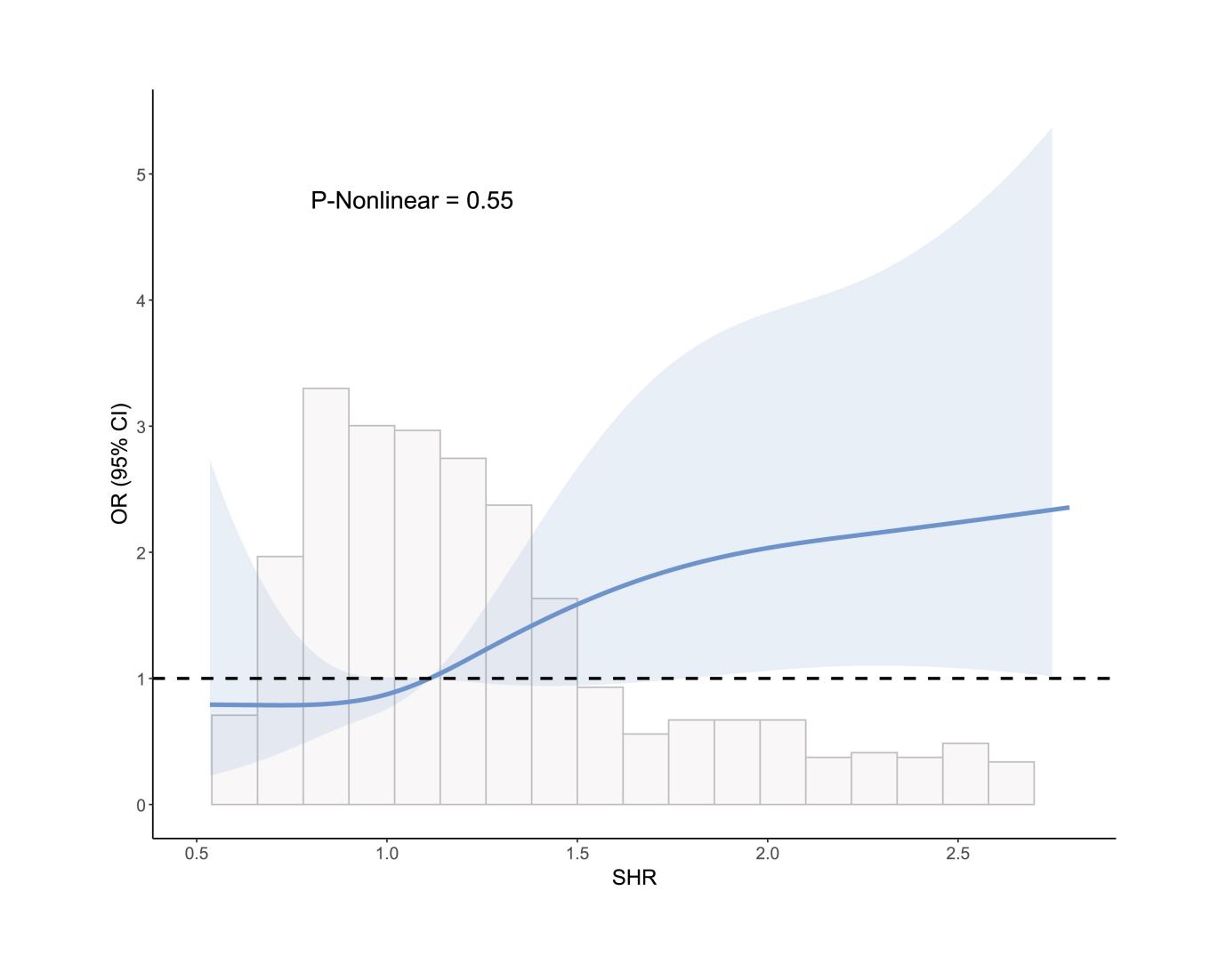
**

Supplement: S2 Fig — (DOCX) [file pone.0323085.s002.docx]
