# Supplementary material for: Association between stress hyperglycemia ratio and poor outcomes in Trauma surgery ICU patients
Source: PLoS One. 2025 May 9;20(5):e0323085. doi: 10.1371/journal.pone.0323085 (PMC12063898; doi:10.1371/journal.pone.0323085)
Supplement: S1 Table — (DOCX) [file pone.0323085.s003.docx]

| **S1 Table**. **Covariance analysis between variables.** | |
| --- | --- |
| **Variables** | **VIF** |
| Age | 1.637 |
| Gender | 1.230 |
| Race | 1.161 |
| Weight | 1.340 |
| Heart rate | 1.462 |
| MBP | 1.434 |
| Temperature | 1.224 |
| SpO2 | 1.361 |
| Hemoglobin | 1.572 |
| Platelet | 1.091 |
| Aniongap | 1.289 |
| Creatinine | 2.142 |
| BUN | 2.256 |
| Sodium | 1.204 |
| Potassium | 1.250 |
| INR | 1.216 |
| Hypertension | 1.269 |
| Diabetes | 1.349 |
| Morphine | 1.147 |
| Fentanyl | 1.500 |
| Dexmedetomidine | 1.115 |
| Antibiotic | 1.441 |
| Insulin | 1.342 |
| Glucocorticoid | 1.141 |
| GCS | 1.140 |
| BUN, blood urea nitrogen; GCS, glasgow coma scale; INR, international normalized ratio; MBP, mean blood pressure; SpO2, pulse blood oxygen saturation; VIF, variance inflation factor. | |
