# Supplementary material for: Association between stress hyperglycemia ratio and poor outcomes in Trauma surgery ICU patients
Source: PLoS One. 2025 May 9;20(5):e0323085. doi: 10.1371/journal.pone.0323085 (PMC12063898; doi:10.1371/journal.pone.0323085)
Supplement: S2 Table — (DOCX) [file pone.0323085.s004.docx]

| **S2 Table. Baseline characteristics according to SHR tertiles.** | | | | |
| --- | --- | --- | --- | --- |
| **Variables** | **Total (n = 569)** | **T1-2  (n = 379)** | **T3  (n = 190)** | ***P-*value** |
|  |  |  |  |  |
| **Demographics** |  |  |  |  |
| Age, years | 66 (54, 76) | 67 (56, 77) | 62 (51, 72) | <.001 |
| Male, n(%) | 328 (57.6) | 213 (56.2) | 115 (60.5) | 0.325 |
| Race, n(%) |  |  |  | 0.002 |
| White | 327 (57.5) | 235 (62.0) | 92 (48.4) |  |
| Other | 242 (42.5) | 144 (38.0) | 98 (51.6) |  |
| Weight, kg | 80.5 (66.0, 97.5) | 80.1 (65.4, 95.7) | 81.0 (68.1, 99.9) | 0.111 |
| **Vital signs** |  |  |  |  |
| Heart rate, bpm | 85 (74, 96) | 82 (72, 92) | 89 (77, 103) | <.001 |
| MBP, mmHg | 82 (74, 90) | 82 (75, 91) | 81 (74, 90) | 0.419 |
| Temperature, ℃ | 36.9 (36.7, 37.3) | 37.0 (36.7, 37.3) | 36.9 (36.7, 37.2) | 0.130 |
| SpO2, % | 98 (96, 99) | 98 (96, 99) | 98 (96, 99) | 0.013 |
| **Laboratory tests** |  |  |  |  |
| Hemoglobin, g/dL | 11.1 (9.4, 12.7) | 11.3 (9.5, 12.9) | 10.8 (9.2, 12.3) | 0.016 |
| Platelet, K/μL | 208 (156, 280) | 215 (162, 284) | 198 (150, 264) | 0.083 |
| Anion gap, mmol/L | 14 (12, 16) | 14 (12, 15) | 14 (12, 16) | 0.002 |
| Creatinine, mg/dL | 1.0 (0.7, 1.3) | 0.9 (0.7, 1.3) | 1.1 (0.8, 1.5) | 0.022 |
| BUN, mg/dL | 18 (13, 26) | 17 (13, 25) | 19 (13, 29) | 0.180 |
| Sodium, mmol/L | 139 (136, 141) | 139 (136, 141) | 139 (136, 142) | 0.661 |
| Potassium, mmol/L | 4.1 (3.8, 4.5) | 4.0 (3.7, 4.4) | 4.2 (3.8, 4.8) | <.001 |
| INR | 1.2 (1.1, 1.4) | 1.2 (1.1, 1.4) | 1.3 (1.1, 1.5) | 0.058 |
| Glucose, mg/dL | 140 (113, 191) | 123 (103, 149) | 200 (164, 266) | <.001 |
| HbA1c, % | 5.9 (5.5, 7.0) | 6.0 (5.6, 7.3) | 5.7 (5.3, 6.7) | <.001 |
| SHR | 1.11 (0.89, 1.37) | 0.97 (0.82, 1.11) | 1.55 (1.37, 1.99) | <.001 |
| **Comorbidities, n (%)** |  |  |  |  |
| Hypertension | 258 (45.3) | 184 (48.6) | 74 (39.0) | 0.030 |
| Diabetes | 234 (41.1) | 157 (41.4) | 77 (40.5) | 0.837 |
| **Medications, n (%)** |  |  |  |  |
| Morphine | 166 (29.2) | 96 (25.3) | 70 (36.8) | 0.004 |
| Fentanyl | 201 (35.3) | 114 (30.1) | 87 (45.8) | <.001 |
| Dexmedetomidine | 28 (4.9) | 21 (5.5) | 7 (3.7) | 0.334 |
| Antibiotic | 262 (46.1) | 152 (40.1) | 110 (57.9) | <.001 |
| Insulin | 241 (42.4) | 133 (35.1) | 108 (56.8) | <.001 |
| Glucocorticoid | 77 (13.5) | 48 (12.7) | 29 (15.3) | 0.393 |
| **Clinical scores** |  |  |  |  |
| GCS | 15 (13, 15) | 14 (12, 15) | 15 (14, 15) | <.001 |
| SOFA | 3 (2, 6) | 3 (2, 5) | 4 (2, 7) | <.001 |
| SAPS II | 33 (26, 42) | 32 (26, 42) | 36 (29, 44) | 0.009 |
| APS III | 41 (31, 56) | 38 (29, 52) | 46 (35, 61) | <.001 |
| OASIS | 32 (26, 37) | 31 (26, 37) | 34 (27, 40) | 0.015 |
| **Outcomes, n (%)** |  |  |  |  |
| 30-day mortality | 118 (20.7) | 58 (15.3) | 60 (31.6) | <.001 |
| 365-day mortality | 185 (32.5) | 104 (27.4) | 81 (42.6) | <.001 |
| Hospital mortality | 97 (17.1) | 48 (12.7) | 49 (25.8) | <.001 |
| APS III, acute physiology score III; BUN, blood urea nitrogen; GCS, glasgow coma scale; HbA1c, hemoglobin A1c; INR, international normalized ratio; MBP, mean blood pressure; OASIS, oxford acute severity of illness score; SAPS II, Simplified Acute Physiology Score II; SHR, stress hyperglycemia ratio; SOFA, sequential organ failure assessment; SpO2, pulse blood oxygen saturation. | | | | |
