# Supplementary material for: Association between stress hyperglycemia ratio and poor outcomes in Trauma surgery ICU patients
Source: PLoS One. 2025 May 9;20(5):e0323085. doi: 10.1371/journal.pone.0323085 (PMC12063898; doi:10.1371/journal.pone.0323085)
Supplement: S3 Table — (DOCX) [file pone.0323085.s005.docx]

| **S3 Table. Baseline characteristics of the 30-day survivor and 30-day mortality group.** | | | | |
| --- | --- | --- | --- | --- |
| **Variables** | **Total (n = 569)** | **30-day survivor (n = 451)** | **30-day mortality (n = 118)** | ***P-*value** |
|  |  |  |  |  |
| **Demographics** |  |  |  |  |
| Age, years | 66 (54, 76) | 65 (54, 74) | 66 (48, 79) | 0.966 |
| Male, n(%) | 328 (57.6) | 258 (57.2) | 70 (59.3) | 0.679 |
| Race, n(%) |  |  |  | <.001 |
| White | 327 (57.5) | 275 (61.0) | 52 (44.1) |  |
| Other | 242 (42.5) | 176 (39.0) | 66 (55.9) |  |
| Weight, kg | 80.5 (66.0, 97.5) | 82.2 (66.6, 100.0) | 76.9 (64.4, 87.4) | 0.006 |
| **Vital signs** |  |  |  |  |
| Heart rate, bpm | 85 (74, 96) | 83 (73, 94) | 90 (75, 104) | 0.008 |
| MBP, mmHg | 82 (74, 90) | 82 (75, 91) | 82 (74, 90) | 0.804 |
| Temperature, ℃ | 36.9 (36.7, 37.3) | 37.0 (36.7, 37.2) | 36.9 (36.5, 37.3) | 0.051 |
| SpO2, % | 98 (96, 99) | 98 (96, 99) | 98 (97, 99) | 0.002 |
| **Laboratory tests** |  |  |  |  |
| Hemoglobin, g/dL | 11.1 (9.4, 12.7) | 11.2 (9.4, 12.7) | 10.9 (9.5, 12.8) | 0.775 |
| Platelet, K/μL | 208 (156, 280) | 219 (166, 293) | 176 (125, 219) | <.001 |
| Anion gap, mmol/L | 14 (12, 16) | 14 (12, 16) | 14 (12, 17) | 0.004 |
| Creatinine, mg/dL | 1.0 (0.7, 1.3) | 0.9 (0.7, 1.3) | 1.1 (0.8, 1.8) | 0.002 |
| BUN, mg/dL | 18 (13, 26) | 17 (12, 25) | 20 (14, 31) | 0.013 |
| Sodium, mmol/L | 139 (136, 141) | 138 (136, 141) | 141 (137, 145) | <.001 |
| Potassium, mmol/L | 4.1 (3.8, 4.5) | 4.1 (3.7, 4.5) | 4.1 (3.8, 4.6) | 0.212 |
| INR | 1.2 (1.1, 1.4) | 1.2 (1.1, 1.4) | 1.3 (1.1, 1.5) | 0.005 |
| Glucose, mg/dL | 140 (113, 191) | 137 (111, 186) | 155 (122, 210) | 0.057 |
| HbA1c, % | 5.9 (5.5, 7.0) | 6.0 (5.5, 7.5) | 5.6 (5.2, 6.1) | <.001 |
| SHR | 1.11 (0.89, 1.37) | 1.08 (0.88, 1.32) | 1.28 (0.99, 1.80) | <.001 |
| **Comorbidities** |  |  |  |  |
| Hypertension, n (%) | 258 (45.3) | 220 (48.8) | 38 (32.2) | 0.001 |
| Diabetes, n (%) | 234 (41.1) | 204 (45.2) | 30 (25.4) | <.001 |
| **Medications, n (%)** |  |  |  |  |
| Morphine | 166 (29.2) | 149 (33.0) | 17 (14.4) | <.001 |
| Fentanyl | 201 (35.3) | 151 (33.5) | 50 (42.4) | 0.072 |
| Dexmedetomidine | 28 (4.9) | 23 (5.1) | 5 (4.2) | 0.700 |
| Antibiotic | 262 (46.1) | 197 (43.7) | 65 (55.1) | 0.027 |
| Insulin | 241 (42.4) | 181 (40.1) | 60 (50.9) | 0.036 |
| Glucocorticoid | 77 (13.5) | 57 (12.6) | 20 (17.0) | 0.223 |
| **Clinical scores** |  |  |  |  |
| GCS | 15 (13, 15) | 14 (13, 15) | 15 (11, 15) | 0.640 |
| SOFA | 3 (2, 6) | 3 (2, 5) | 6 (4, 9) | <.001 |
| SAPS II | 33 (26, 42) | 32 (25, 40) | 41 (33, 52) | <.001 |
| APS III | 41 (31, 56) | 38 (29, 51) | 56 (43, 74) | <.001 |
| OASIS | 32 (26, 37) | 30 (25, 36) | 37 (33, 42) | <.001 |
| APS III, acute physiology score III; BUN, blood urea nitrogen; GCS, glasgow coma scale; HbA1c, hemoglobin A1c; INR, international normalized ratio; MBP, mean blood pressure; OASIS, oxford acute severity of illness score; SAPS II, Simplified Acute Physiology Score II; SHR, stress hyperglycemia ratio; SOFA, sequential organ failure assessment; SpO2, pulse blood oxygen saturation. | | | | |
