# Supplementary material for: Association between stress hyperglycemia ratio and poor outcomes in Trauma surgery ICU patients
Source: PLoS One. 2025 May 9;20(5):e0323085. doi: 10.1371/journal.pone.0323085 (PMC12063898; doi:10.1371/journal.pone.0323085)
Supplement: S4 Table — (DOCX) [file pone.0323085.s006.docx]

| **S4 Table. Univariate and multivariate logistic regression analysis of factors influencing hospital mortality.** | | | | | |
| --- | --- | --- | --- | --- | --- |
|  | **Univariate analysis** | |  | **Multivariate analysis** | |
| **Variables** | ***P*** | **OR (95% CI)** |  | ***P*** | **OR (95% CI)** |
| SHR | <.001 | 2.11 (1.47-3.04) |  | 0.005 | 1.83 (1.20-2.80) |
| Age | 0.832 | 1.00 (0.99-1.01) |  |  |  |
| Gender, Male | 0.836 | 0.95 (0.61-1.48) |  |  |  |
| Race, White | 0.004 | 0.53 (0.34-0.82) |  | 0.105 | 0.65 (0.39-1.09) |
| Weight | 0.021 | 0.99 (0.98-0.99) |  | 0.020 | 0.99 (0.97-0.99) |
| Heart rate | 0.023 | 1.02 (1.01-1.03) |  |  |  |
| MBP | 0.159 | 0.99 (0.97-1.01) |  |  |  |
| Temperature | 0.038 | 0.64 (0.43-0.98) |  | 0.134 | 0.72 (0.47-1.11) |
| SpO2 | 0.024 | 1.16 (1.02-1.32) |  |  |  |
| Hemoglobin | 0.981 | 1.00 (0.91-1.11) |  |  |  |
| Platelet | <.001 | 0.99 (0.99-0.99) |  | <.001 | 0.99 (0.99-0.99) |
| Anion gap | 0.001 | 1.10 (1.04-1.17) |  |  |  |
| Creatinine | 0.011 | 1.20 (1.04-1.39) |  |  |  |
| BUN | <.001 | 1.02 (1.01-1.03) |  | 0.001 | 1.02 (1.01-1.03) |
| Sodium | <.001 | 1.13 (1.08-1.19) |  | <.001 | 1.10 (1.04-1.16) |
| Potassium | 0.183 | 1.24 (0.90-1.71) |  |  |  |
| INR | 0.028 | 1.60 (1.05-2.43) |  | 0.052 | 1.58 (1.00-2.49) |
| Hypertension | 0.004 | 0.51 (0.32-0.81) |  |  |  |
| Diabetes | 0.008 | 0.52 (0.33-0.84) |  |  |  |
| Morphine | <.001 | 0.29 (0.15-0.55) |  | <.001 | 0.29 (0.14-0.58) |
| Fentanyl | 0.002 | 2.04 (1.31-3.18) |  | 0.010 | 2.00 (1.18-3.39) |
| Dexmedetomidine | 0.691 | 0.80 (0.27-2.37) |  |  |  |
| Antibiotic | 0.006 | 1.86 (1.19-2.89) |  |  |  |
| Insulin | 0.026 | 1.65 (1.06-2.55) |  |  |  |
| Glucocorticoid | 0.115 | 1.59 (0.89-2.85) |  |  |  |
| GCS | 0.125 | 0.95 (0.88-1.02) |  |  |  |
| BUN, blood urea nitrogen; GCS, glasgow coma scale; INR, international normalized ratio; MBP, mean blood pressure; SHR, stress hyperglycemia ratio; SpO2, pulse blood oxygen saturation. | | | | | |
