# Supplementary material for: Association between stress hyperglycemia ratio and poor outcomes in Trauma surgery ICU patients
Source: PLoS One. 2025 May 9;20(5):e0323085. doi: 10.1371/journal.pone.0323085 (PMC12063898; doi:10.1371/journal.pone.0323085)
Supplement: S5 Table — (DOCX) [file pone.0323085.s007.docx]

| **S5 Table. Incremental effect of SHR in predicting hospital mortality.** | | | | |
| --- | --- | --- | --- | --- |
| **Model 1** | **AUC (95% CI)** | **Model 2** | **AUC (95% CI)** | ***P* for comparison** |
| Hospital mortality |  |  |  |  |
| SOFA | 0.760 (0.708-0.812) | + SHR | 0.773 (0.723-0.823) | 0.025 |
| SAPS II | 0.742 (0.691-0.792) | + SHR | 0.769 (0.722-0.815) | 0.007 |
| APS III | 0.721 (0.665-0.777) | + SHR | 0.743 (0.689-0.796) | 0.014 |
| OASIS | 0.730 (0.678-0.782) | + SHR | 0.756 (0.705-0.806) | 0.016 |
| APS III, acute physiology score III; AUC, area under the curve; OASIS, oxford acute severity of illness score; SAPS II, simplified acute physiology score II; SHR, stress hyperglycemia ratio; SOFA, sequential organ failure assessment. | | | | |
